# Exploring Immune Cell Infiltration and Small Molecule Compounds for Ulcerative Colitis Treatment

**DOI:** 10.3390/genes15121548

**Published:** 2024-11-29

**Authors:** Yi Lu, Dongqing Lu, Chujie Li, Luping Chen

**Affiliations:** 1 Shanghai Tufeng Pharmaceutical Technology Co., Ltd., Shanghai 201203, China; 2 Jiangsu Kanion Pharmaceutical Co., Ltd., Lianyungang 222001, China; 3 Department of Traditional Chinese Medicine, Beicai Community Health Service Center of Pudong New District, 271 Lianyuan Road, Pudong New District, Shanghai 201024, China; 4 Department of Pharmacology and Personalized Medicine, School of Nutrition and Translational Research in Metabolism (NUTRIM), Maastricht University, 6200 MD Maastricht, The Netherlands; 5 The M-Lab., Department of Precision Medicine, GROW—Research Institute for Oncology and Repro-Duction, Maastricht University, 6200 MD Maastricht, The Netherlands; 6 Department of Pharmacology and Toxicology, School of Nutrition and Translational Research in Metabolism (NUTRIM), Maastricht University, 6200 MD Maastricht, The Netherlands

**Keywords:** bioinformatics, differentially expressed genes, gene chip, potential therapeutic drugs, ulcerative colitis

## Abstract

Background/Objectives: Ulcerative colitis (UC) is a chronic inflammatory bowel disease (IBD) with a relapsing nature and complex etiology. Bioinformatics analysis has been widely applied to investigate various diseases. This study aimed to identify crucial differentially expressed genes (DEGs) and explore potential therapeutic agents for UC. Methods: The GSE47908 and GSE55306 colon tissue transcriptome gene datasets were downloaded from the Gene Expression Omnibus-NCBI (GEO) database. GEO2R and Gene Set Enrichment Analysis (GSEA) were used to screen for DEGs in patients with UC compared to the normal population based on weighted gene co-expression network analysis (WGCNA). GO-BP analysis and KEGG enrichment analysis were performed on the intersecting differential genes via the Metascape website, while hub genes were analyzed by STRING11.0 and Cytoscape3.7.1. The expression of hub genes was verified in the dataset GSE38713 colon tissue specimens. Finally, the gene expression profiles of the validation set were analyzed by immuno-infiltration through the ImmuCellAI online tool, and the CMap database was used to screen for negatively correlated small molecule compounds. Results: A total of 595 and 926 genes were screened by analysis of GSE47908 and GSE55306 datasets, respectively. Combined WGCNA hub module intersection yielded 12 hub genes (CXCL8, IL1β, CXCL1, CCL20, CXCL2, CXCR2, LCN2, SELL, AGT, LILRB3, MMP3, IDO1) associated with the pathogenesis of UC. GSEA analysis yielded intersecting pathways for both datasets (colorectal cancer pathway, base excision repair, cell cycle, apoptosis). GO-BP and KEGG enrichment analyses were performed to obtain key biological processes (inflammatory response, response to bacteria, leukocyte activation involved in the immune response, leukocyte–cell adhesion, apoptosis, positive regulation of immune effector processes) and key signaling pathways (cytokine–cytokine receptor interactions, IBD, NOD-like receptor signaling pathways). The immune cell infiltration analysis suggested that the incidence of UC was mainly related to the increase in CD4+T cells, depletion of T cells, T follicular helper cells, natural killer cells, γδ T cells and the decrease in CD8 naive T cells, helper T cells 17 and effector T cells. The CMap database results showed that small molecule compounds such as vorinostat, roxarsone, and wortmannin may be therapeutic candidates for UC. Conclusions: This study not only aids in early prediction and prevention but also provides novel insights into the pathogenesis and treatment of UC.

## 1. Introduction

Ulcerative colitis (UC) is defined medically as a chronic, nonspecific and recurrent inflammatory bowel disease (IBD) involving the rectum and colonic mucosa [1]. At present, the cause of UC remains elusive. It is generally believed that several factors such as severe intoxication [2], improper diet [3], stress [4] and genetic factors [5] contribute to its development. The typical pathological feature of UC involves mucosal and submucosal inflammation, ulcerations, and crypt abscesses [6]. According to its severity, UC can be classified into three types: mild, moderate and severe [7]. According to the literature, UC can cause perforation, fistula, cancerous changes, pseudopolyp formation, toxic megacolon, and stenosis disease [8,9]. A meta-analysis pointed out that the probability of UC patients suffering from colon cancer after 10 years, 20 years and 30 years was 1.6%, 8.3% and 18.4% in turn [10]. Due to the unclear pathogenesis and pathogenic factors of UC, the study of pathogenesis is particularly important to develop effective treatment methods.

Experimental diagnostics and clinical treatments for UC are rapidly advancing due to a better understanding of the disease. A recent study has highlighted the importance of identifying clinical biomarkers that can guide treatment decisions and improve patient outcomes [11]. For instance, the integration of bioinformatics into the diagnostic process has shown promise in characterizing the molecular phenotype of UC, which can help predict disease course and response to treatment [12]. Current therapeutic strategies include the use of biological agents, such as anti-TNF therapies and integrin blockers, which have significantly improved clinical management [13]. Additionally, new therapeutic modalities are being explored, including small molecule inhibitors and novel biologics targeting specific cytokines and immune pathways [14]. The potential of personalized medicine in UC management is also gaining traction, with ongoing research aimed at developing predictive biomarkers that can tailor treatments to individual patient profiles [15]. In brief, the current experimental diagnostic and clinical treatments for UC are distinguished by a multifaceted approach that incorporates traditional therapies with pioneering strategies informed by bioinformatics and molecular research.

Data mining, genomic sequencing, gene chips, and bioinformatics have become efficient tools for screening for differentially expressed genes (DEGs) [16]. Based on the Gene Expression Omnibus (GEO) public database, GEO2R, Gene Set Enrichment Analysis (GSEA) combined with weighted gene co-expression network analysis (WGCNA) and other bioinformatics analysis methods were used to screen and identify key genes and key pathways related to UC [17]. Key genes were confirmed using gene chip analysis, immunoinfiltration in UC colon mucosa was assessed with ImmuCellAI abundance, and potential therapeutic small molecule compounds were identified through the CMap database, offering new treatment options for UC.

## 2. Materials and Methods

### 2.1. Microarray Data Analysis

In this study, GSE47908, GSE53306 and validation dataset GSE38713 were retrieved and obtained from the GEO database (https://www.ncbi.nlm.nih.gov/ (accessed on 1 June 2021)). The GSE47908 contains 15 normal samples and 20 UC samples (normal samples: GSM1162227–GSM1162241; UC samples: GSM1162248–GSM1162267). The GSE53306 contains 12 normal samples and 16 UC samples (normal samples: GSM1289196–GSM1289207; UC samples: GSM1289168–GSM1289183). The validation set GSE38713 contains 13 normal samples and 15 UC samples (normal sample: GSM948550–GSM948562; UC sample: GSM948571–GSM948592). All samples are human colonic mucosal tissues, excluding samples treated with immunosuppressants, 5-aminosalicylic acid and samples with inactive UC. In Table 1, we listed all the information on the screened GSE datasets. The probe names were converted into Gene Symbols via the Bioconductor Affy package for the R language, and the expression data were normalized to each other based on the RMA algorithm. DEGs were screened using GEO2R with |Log2FoldChange| > 1 and *p* < 0.05.

### 2.2. Gene Set Enrichment Analysis (GSEA)

We prepared expression datasets (GSE47908 and GSE53306), phenotypic data, and functional gene sets (c2.cp.kegg.v7.4.symbols.gmt) files. We set the software operation parameter as “No collapse”, number of permutations to “500”, permutation to “Phenotype”, and used GSEA_4.1.0 to analyze the above files. Enriched pathways were screened based on NES (corrected enrichment score) and NOM *p*-value. Pathways common to the two expression datasets were then presented as results.

### 2.3. WGCNA Analysis

Matrix data of transcriptome expression profiles were prepared, samples were clustered, soft-threshold β was calculated, and scale-free networks were constructed using the R software WGCNA package and Limma package. The association between genes/modules and UC was evaluated by the significance of genes and modules, and the genes within the two groups of modules with the greatest association were selected as differential genes.

### 2.4. Investigation of Hub Genes

Hub genes refer to genes that play a pivotal role in biological signaling pathway activation. The degree values of the hub genes in the protein–protein interaction (PPI) network were rather high. We analyzed GSE47908 and GSE55306 by GEO2R online software and screened to obtain genes that were significantly altered in UC patients, labeled as “GSE47908-D” and “GSE55306-D”, respectively.

### 2.5. Biological Enrichment Analysis of Intersecting Differential Genes

Based on the results of the WGCNA analysis, Module Membership (MM) and Gene Significance (GS) values of different modules were calculated. The parameters |MM| > 0.8 and |GS| > 0.5 were set to select the DEGs with high correlation with the hub module, which were recorded as “GSE47908-W” and “GSE55306-W”. The core DEGs were mapped by the online Venn tool, and then the top hub genes were screened by STRING-PPI-Cytoscape (3.7.1) coupled with the cytoHubba plugin based on the degree mode for subsequent analyses. The 102 intersecting DEGs were also analyzed for biological process (GO-BP) and KEGG pathway enrichment using the Metascape (https://metascape.org, (accessed on 1 June 2021)).

### 2.6. Analysis of Hub Genes Expression in Validation Set

Hub genes were screened and analyzed via STRING-PPI-Cytoscape. The validation set GeneChip GSE38713 was downloaded from the GEO database, which included a total of 43 adults (13 normal samples, 15 samples with active UC, and 15 samples with remission and uninvolved mucosal tissues) with expression profiling at the transcriptome level of the colonic tissues. After the exclusion of the 15 samples with UC in remission and uninvolved mucosa, the expression of hub genes associated with UC pathogenesis was analyzed in the colonic mucosal tissues of the normal and UC groups.

### 2.7. Immune Cell Infiltration Analysis

The gene expression matrix and grouping information of the validation set GSE38713 were prepared and uploaded to the ImmuCellAI online tool (http://bioinfo.life.hust.edu.cn/ImmuCellAI#!/analysis (accessed on 7 June 2021)). This tool was used to predict the abundance of 24 immune cell types such as T cells, NK cells and DC cells in the samples, and to analyze the differences in immune cell infiltration of colonic tissues in the normal and UC groups.

### 2.8. Screening Small Molecule Compounds

The top 1000 genes with large expression differences in the validation set GeneChip GSE38713 were divided into two groups, up-regulated and down-regulated. These genes were entered into the CMap (http://www.broad.mit.edu/CMap (accessed on 9 June 2021)) database in the form of Query Signature file format. The relevant small molecule compounds were obtained by comparing the UC differential gene profiles with the reference gene expression profiles in the database. The results were sorted by score size to filter out negatively correlated small molecule compounds.

## 3. Results

### 3.1. DEGs Between UC Samples and Normal Samples

**Figure 1:**
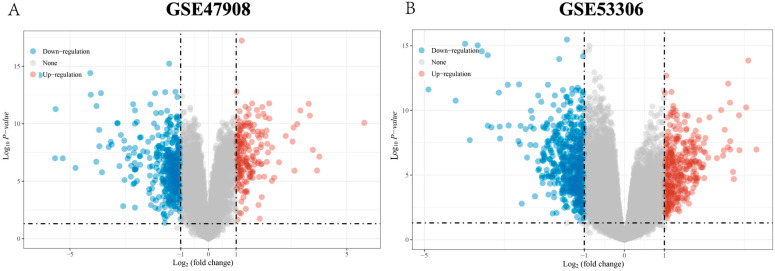
DEGs in datasets of colon tissue samples. (**A**) Volcano of GSE47908. (**B**) Volcano of GSE53306.

The normalization of the data of GSE47908 and GSE55306 expression matrices was performed with R software. We screened the DEGs based on the conditions of |Log2FoldChange| > 1 and *p* < 0.05 for subsequent analysis, and the results are shown in Figure 1. A total of 595 significant DEGs were screened from the GSE47908 microarrays, of which 200 were up-regulated genes, and 295 were down-regulated genes (Figure 1A). A total of 926 DEGs were screened from GSE53306, of which 346 were up-regulated and 580 were down-regulated (Figure 1B). 

### 3.2. WGCNA Analysis and Screening of Genes Within the Hub Module

**Figure 2:**
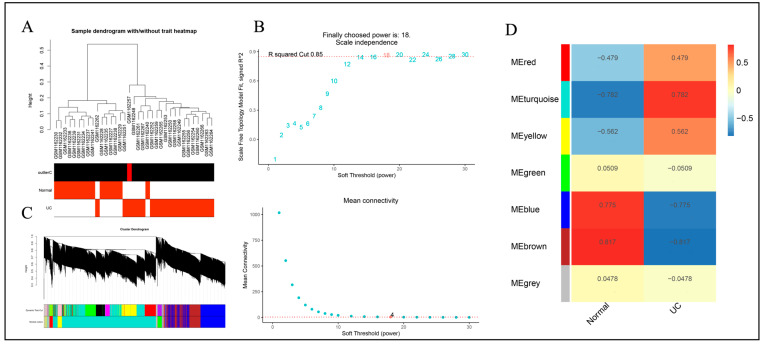
Analysis of WGCNA co-expression module based on GSE47908 gene chip. (**A**) Sample cluster analysis. (**B**) Analysis of the scale-free fit index and the mean connectivity for various soft-thresholding powers (β = 18). (**C**) Dendrogram of genes clustered based on the dissimilarity measure. (**D**) Heatmap of the correlation between different modules with UC.

**Figure 3:**
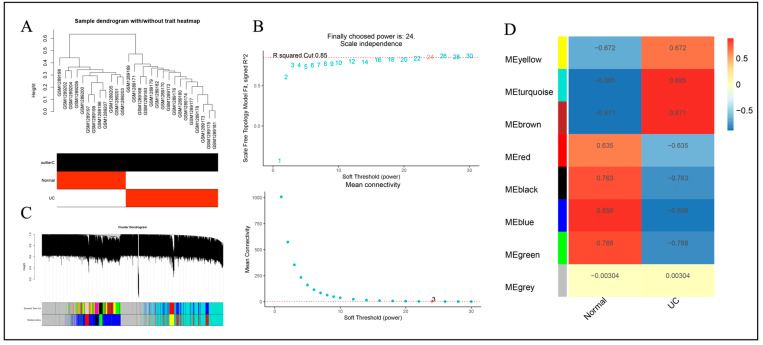
Analysis of WGCNA co-expression module based on GSE53306 gene chip. (**A**) Sample cluster analysis. (**B**) Analysis of the scale-free fit index and the mean connectivity for various soft-thresholding powers (β = 24). (**C**) Dendrogram of genes clustered based on the dissimilarity measure. (**D**) Heatmap of the correlation between different modules with UC.

The samples of GSE47908 and GSE53306 chips were clustered by R software separately, and the samples of both chips showed a better clustering trend (Figure 2A). The chip soft thresholds β (GSE47908) = 18 and β (GSE53306) = 24 were calculated by R software. Gene modules were constructed based on soft thresholds (Figure 2B). In total, 7 gene modules were obtained by dynamic shear tree. The results of associations of different modules with UC were presented in the form of heatmaps (Figure 2C,D). The module MEturquoise (r = 0.782, *p* < 0.05), which was significantly positively correlated with UC, and the negatively correlated module MEbrown (r = −0.817, *p* < 0.05) were selected. Genes within the two modules were integrated and labeled as GSE47908-W for subsequent analysis. The GSE53306 gene chip was analyzed similarly to obtain the hub modules MEturquoise (r = 0.885, *p* < 0.05) and MEblue (r = −0.856, *p* = 0.05). Genes within the two modules were integrated and labeled as GSE55306-W for subsequent analysis (Figure 3).

### 3.3. Screening Hub Genes Associate with UC

**Figure 4:**
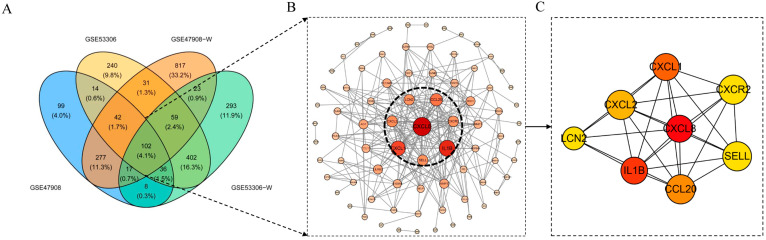
Screening of hub genes. (**A**) Venn diagram of intersection DEGs. (**B**) PPI network construction. (**C**) Hub genes screening based on degree.

The common differential gene intersections of GSE47908-D, GSE53306-D, GSE47908-W, and GSE53306-W were mapped on a Venn diagram. In total, 102 intersecting genes including IL4, NOD2, and IL1β were screened (Figure 4A). PPI analysis of the intersecting genes was done by STRING-Cytoscape (Figure 4B). Screening in degree mode in Cyto Hubba plugin yielded 12 pivotal genes with high scores: CXCL8, IL1β, CXCL1, CCL20, CXCL2, CXCR2, LCN2, SELL, AGT, LILRB3, MMP3, IDO1 (Figure 4C).

**Figure 5:**
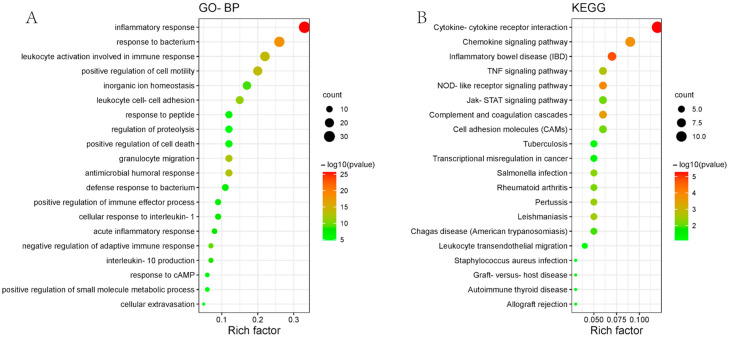
Enrichment analysis of core targets through GO and KEGG. (**A**) GO-BP pathway enrichment analysis. (**B**) KEGG pathway enrichment analysis.

In total, 102 significantly altered intersecting genes in ulcerative colitis patients were selected for the GO−BP and KEGG pathway enrichment analyses. The results suggested that on GO−BP, the intersecting genes were mainly enriched on biological processes such as inflammatory response, response to bacteria, leukocyte activation involved in immune response, leukocyte cell–cell adhesion, and positive regulation of cell motility (Figure 5A). On the KEGG pathway, the intersection genes were mainly enriched on signaling pathways such as cytokine–cytokine receptor interaction, inflammatory bowel disease (IBD), and NOD-like receptor signaling pathway (Figure 5B). The pathway information is listed in Table 2.

### 3.4. Expression Analysis of Hub Genes in Validation Dataset of UC Patients

**Figure 6:**
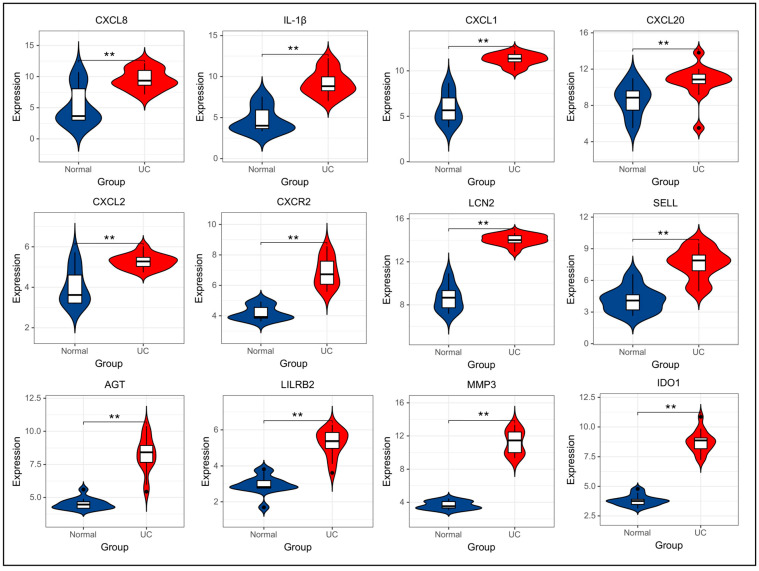
Top 12 hub gene expression in the GSE38713 ulcerative colitis dataset.Significance: **, *p* < 0.01.

The validation dataset GSE38713 was obtained from the GEO database. Baseline information of the subjects such as gender, age, etc., was recorded for each sample. Based on this dataset, we analyzed the expression of the 12 hub genes in this dataset to verify the findings accuracy of the current study. The results showed that the expression of 12 hub genes was significantly upregulated in the colonic tissues of UC patients (Figure 6).

### 3.5. GSEA Analysis

**Figure 7:**
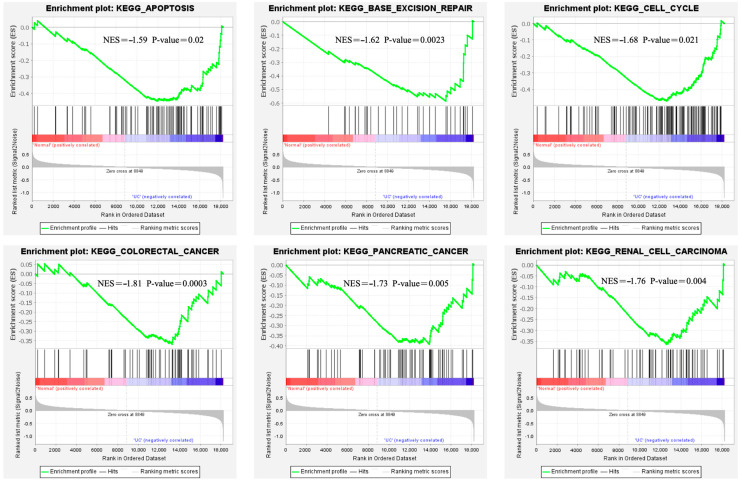
Signaling pathways in the GSE47908 and GSE55306 datasets via GSEA analysis.

We normalized matrix data for GSE47908 and GSE55306. Expression profiles were obtained for 35 samples containing 20,549 genes and 28 samples containing 18,234 genes, respectively. The intersection of the two microarrays was obtained using the KEGG pathway gene set as a classification criterion, including the base excision repair pathway (NES = −1.62, *p* = 0.0023); cell cycle pathway (NES = −1.76, *p* = 0.004); colorectal cancer pathway (NES = −1.81, *p* = 0.0003); pancreatic cancer pathway (NES = −1.73, *p* = 0.005); apoptosis pathway (NES = −1.59, *p* = 0.02) (Figure 7).

### 3.6. Differential Analysis of Immune Infiltrates Between UC and Normal Group

**Figure 8:**
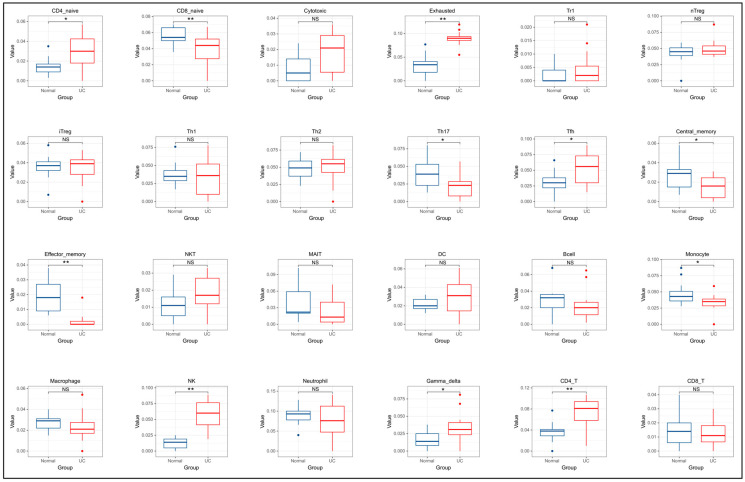
Immune cell infiltration in ulcerative colitis and control groups. Significance: NS, *p* > 0.05; *, *p* < 0.05; **, *p* < 0.01.

As shown in Figure 8, the differential analysis of immune infiltrating cells between UC and normal groups was performed by box plots, and *p* < 0.05 was considered as a significant difference. It was concluded that CD4 naive T cells (*p* = 0.02), depleted T cells (*p* = 0.00), T follicular helper (Tfh) cells (*p* = 0.02), natural killer (NK) cells (*p* = 0.00), γ delta T cells (γ_delta) (*p* = 0.02), and CD4 T cells (*p* = 0.00) were significantly higher in the UC group. In addition, CD8 naive T cells (*p* = 0.01), helper T cells17 (*p* = 0.02), effector memory T cells (effector memory T cells) (*p* = 0.00), and monocytes (monocytes) (*p* = 0.02) were significantly reduced in the colonic mucosal tissue of UC.

### 3.7. Screening of Therapeutic Candidates for UC

The CMap database was used to screen the 10 most negatively correlated small molecule compounds with the highest degree of association, as shown in Table 3.

## 4. Discussion

The incidence of UC has increased rapidly with the process of industrialization and the change in dietary habits [18]. Studies have shown that the risk of digestive system cancer among severe UC patients is significantly higher compared to mild and moderate UC patients [19,20,21]. Moreover, the prognosis of UC is poor, and patients are prone to fatigue [22], malnutrition [23] and anemia [24], which seriously affects the quality of life. Current UC treatments include a variety of therapeutic approaches aimed at managing the disease and achieving remission. Traditional treatments, such as mesalamine, remain the core therapy for mild-to-moderate ulcerative colitis (UC), while corticosteroids are crucial for inducing remission in moderate-to-severe flares [25]. Additionally, the exploration of combination therapies and personalized medicine approaches aims to enhance treatment efficacy and minimize adverse effects [26]. For instance, research is being conducted on the use of sphingosine 1-phosphate receptor modulators, such as etrasimod, which have demonstrated clinical benefits in patients with moderately to severely active UC [27]. Moreover, innovative treatment modalities are being investigated, including the targeted delivery of herbal compounds like berberine and tannic acid, which have shown promise in reducing colonic inflammation [28]. As the understanding of UC pathophysiology improves, particularly regarding the role of the microbiome and cell death mechanisms like ferroptosis, new therapeutic avenues are expected to emerge, potentially transforming the management of this chronic condition [29]. Therefore, it is extremely important to study the mechanism and clinical prevention and treatment of UC. With the advancement of gene chip technology, the pathogenesis of diseases can be precisely elucidated through the application of bioinformatics. Concurrently, the extensive data generated by bioinformatics offers novel avenues for drug research and development.

**Figure 9:**
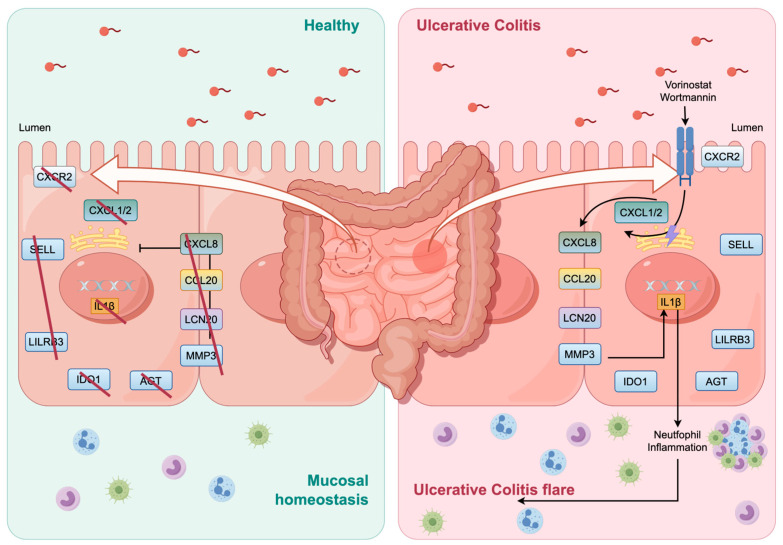
The impact of hub genes expression on the immune cell infiltration in the colonic mucosa with the therapeutic activity of the best drug candidates (vorinostat and wortmannin).

To investigate the microscopic characteristics of pathological tissues in the context of UC, this study elucidated the alterations in gene expression profiles within patients’ colonic tissues at the molecular level. Furthermore, key differential genes and signaling pathways associated with UC were identified and analyzed using bioinformatics approaches, leveraging data from public databases. Through a comprehensive multi-database analysis of genes exhibiting differential expression between healthy and diseased individuals, alongside the enrichment of associated signaling pathways and biological functions, this study aims to elucidate the underlying mechanisms of UC from a holistic perspective. The findings from this study are anticipated to guide the design of subsequent targeted experiments. In this research, transcription gene chip datasets GSE47908 and GSE55306, encompassing colon mucosa tissue from both healthy individuals and patients with ulcerative colitis (UC), were collected for bioinformatics analysis. A total of 102 intersecting differential genes, including 12 pivotal genes (CXCL8, IL1β, CXCL1, CCL20, CXCL2, CXCR2, LCN2, SELL, AGT, LILRB2, MMP3, IDO1), were identified through a combined analysis using GEO2R and Weighted Gene Co-Expression Network Analysis (WGCNA). GO-BP annotation showed that these differential genes were mainly enriched in the inflammatory reaction, leukocyte activation, neutrophil migration, and positive regulation of immune effects. Specifically, CXCL8, CXCL1, and CXCL2, collectively referred to as neutrophil factors, are members of the CXC chemokine family [30]. These chemokines interact with the CXCR2 receptor to facilitate the activation of neutrophils, promoting their migration and adhesion to sites of inflammation [31]. CCL20 is involved in the recruitment of T cells and dendritic cells, thereby enhancing immune responses [32]. Additionally, LCN2 encodes neutrophil gelatinase-associated lipocalin, a critical component of innate immunity and a recognized marker of intestinal inflammation [33]. Furthermore, MMP3 is involved in the degradation of the extracellular matrix during both physiological and pathological processes [34]. Cell surface adhesion molecules encoded by the SELL gene facilitate leukocyte migration to secondary lymphoid organs and sites of inflammation [35]. Additionally, a heme enzyme encoded by the IDO1 gene modulates T cell behavior through the regulation of tryptophan catabolism [36]. AGT is associated with inflammation through the renin–angiotensin system [37]. LILRB3 modifies immune cell signaling, potentially influencing immune dysregulation [38]. Collectively, these processes result in immune cell infiltration, mucosal inflammation, and colonic tissue damage, which are characteristic of ulcerative colitis (UC). The illustration depicts the impact of these genes’ expression on immune cell infiltration in the colonic mucosa, as well as the therapeutic activity of the most promising drug candidates (Figure 9).

Many of the 102 intersection genes identified in the screening possess significant research relevance in the pathogenesis of UC. For instance, S100A9, a component of calprotectin, can induce neutrophil degranulation by activating the MAPK and PI3K/AKT signaling pathways [39]. As members of the aquaporin family, AQP3 and AQP9 are integral to gastrointestinal water transport [40], potentially contributing to symptoms such as diarrhea in UC [41]. Analysis of the validation dataset GSE38713 revealed significant differential expression of all pivotal genes between the two groups under study. Moreover, no significant differences were observed in the expression of intersecting genes between male and female UC patients, corroborating the clinical observation that UC does not exhibit a gender bias. The GSEA conducted on two distinct gene chips indicates a predominant enrichment in KEGG pathways, including base excision repair, cell cycle, apoptosis, and the colorectal cancer pathway. Pertinent studies suggest that alterations in the base excision repair pathway and the colorectal cancer pathway are significant. Specifically, DNA damage induced by persistent oxidative stress is typically rectified by the base excision repair enzyme [42]. However, abnormalities in this enzyme may result in mutations of genes associated with UC, potentially leading to related malignant complications [43]. These findings can provide a theoretical basis for further study on the pathogenesis of UC.

In this study, GO-BP and KEGG analyses were conducted on DEGs in colon tissues from patients with ulcerative colitis and healthy controls, utilizing data from the GEO microarray. The findings indicate that the biological processes associated with these differential genes are intricately linked to immune regulation. The processes in question encompass leukocyte activation, leukocyte-epithelial cell adhesion, neutrophil migration, and the negative regulation of adaptive immunity. These findings provide additional evidence that UC is an immunological disorder marked by the participation of diverse inflammatory cells and mediators. KEGG pathway enrichment analysis revealed that, aside from the inflammatory bowel disease (IBD) pathway, there was significant gene enrichment in the NOD-like receptor and TNF-α signaling pathways. The NOD-like receptor (NLR) is an integral component of the signal transduction ATPase, which is crucial for the regulation of the host’s innate immune response [44]. It initiates the signal transduction pathways of nuclear factor κB (NF-κB) [45] and mitogen-activated protein kinase (MAPK) [46], thereby regulating the activation of inflammatory caspases. This mechanism is pivotal in host–pathogen interactions and inflammatory responses. Additionally, tumor necrosis factor-α (TNF-α) is closely associated with NF-κB [47]. TNF-α is secreted by intestinal epithelial cells and macrophages in the IBD pathway via the Toll-like receptor-NF-κB signaling cascade [48]. This cytokine exerts a direct pro-inflammatory effect, enhances neutrophil phagocytosis [49], and induces pyrexia [50].

To further investigate the role of immune cells in the colonic tissue of patients with UC, the study utilized the ImmuCellAI online tool to screen two groups of colonic gene samples in the validation set. The final results indicated that, in comparison to the normal group, the proportions of naive CD4+ T cells (CD4_native), exhausted T cells (T-Exhausted), follicular helper T cells (Tfh), natural killer cells (NK), and γδ T cells (γ-delta) were significantly elevated in the UC group. Conversely, the proportions of naive CD8+ T cells (CD8_native), memory T cells (memory T), monocytes (monocyte), and helper T cells 17 (Th17) were significantly reduced in the UC colon tissue. These experimental findings were corroborated by fundamental research. Upon infection by an antigen, antigen-specific naive CD4+ T cells become activated, undergoing rapid proliferation and differentiation into memory T cells and effector T cells. However, during chronic infections such as UC, prolonged antigenic stimulation impedes the transformation of naive T cells into antigen-independent memory T cells. Instead, these cells differentiate into a dysfunctional phenotype known as exhausted T cells. These cells represent a subset of T cells characterized by diminished effector function and sustained expression of inhibitory receptors, predominantly observed in a state of T cell dysfunction, rendering them incapable of mounting an immune response to antigens [51]. The disequilibrium between T follicular helper (Tfh) cells, which facilitate antibody production by B cells, and T follicular regulatory (Tfr) cells, which inhibit this process, constitutes a critical mechanism in the progression of UC [52]. Notably, T follicular helper (Tfh) cells may play a beneficial role in attenuating the progression of UC [53]. Research has indicated a significant increase in Th1 and Th2 cell populations within the colonic tissue of UC patients [54]. However, experimental data reveal no substantial differences in T cell populations between the control and UC groups. It is important to consider that the ImmuCellAI tool may exhibit bias in quantifying immune cell numbers, potentially attributable to the limited sample size.

The CMap database currently stands as the most influential repository for expression profiles of active compounds or drugs and their associated genes [55]. It serves as a bioinformatics analysis platform pivotal for drug discovery and elucidating the relationships between genes and diseases. In this study, 10 small molecules exhibiting negative correlation were identified from the database, including wortmannin [56], LY294002 [57], and rapamycin [58], which are known inhibitors of the PI3K/AKT/mTOR pathway. Numerous experimental studies have demonstrated that inhibition of this pathway can ameliorate the pathological state in murine models of UC [59]. Vorinostat [60] and trichostatin A [61], as classical pan-HDAC inhibitors [62], play a crucial role in intestinal barrier protection by reducing the acetylation level of nucleosome histones and inhibiting the expression of NF-κB, thereby down-regulating the levels of inflammatory factors. These findings demonstrate the feasibility of utilizing the CMap database to identify potential therapeutic strategies involving small molecular compounds for the treatment of UC. Simultaneously, the findings demonstrate that inhibitors of the PI3K/AKT/mTOR pathway and HDAC inhibitors serve as intervention molecules capable of reversing inflammatory cytokine levels and restoring the intestinal barrier in UC colon tissue, highlighting their potential as therapeutic agents for UC.

Recent advances have highlighted the potential of artificial intelligence (AI) in bioinformatics and gene therapy, especially in the treatment of complex diseases such as ulcerative colitis (UC). The application of AI can help physicians more accurately assess disease activity and predict treatment response by analyzing large-scale multimodal data [63]. For example, AI-assisted endoscopic techniques have been developed to assess mucosal healing in UC, and studies have shown that AI can significantly improve diagnostic consistency and accuracy [64]. In terms of treatment, the integration of AI may promote the development of personalized medicine, allowing doctors to develop more effective treatment plans according to the patient’s specific situation [65]. For example, AI can analyze genomic, metabolome and microbiome data to help identify the most suitable treatment [66]. Despite the great potential of AI in UC treatment, challenges of data quality, standardization and clinical implementation remain, and further research and guidelines are needed to overcome.

## 5. Conclusions

In summary, this study identified ten pivotal genes, key pathways, and biological processes associated with the pathogenesis of UC through bioinformatics analyses utilizing GEO2R, GSEA, and WGCNA. Additionally, the infiltration patterns of immune cells in UC colon tissue were analyzed using the ImmuCellAI online tool (http://bioinfo.life.hust.edu.cn/ImmuCellAI#!/analysis (accessed on 1 June 2021)). Finally, the therapeutic drugs related to UC were predicted by the CMap small molecule drug database, which will help to further explore the pathogenesis and provide new ideas for clinical diagnosis and treatment of UC. This research provides a comprehensive approach to understanding the disease’s pathogenesis, improving early diagnosis, and developing targeted therapies. This holistic approach aims to enhance patient outcomes and quality of life through tailored medical strategies. Some limitations remain in our study, including small sample sizes, lack of randomization and potential biases, and variability or non-standardization in treatment protocols. In the future, we will incorporate AI tools to further refine our exploration scheme for potential therapeutic targets in UC.

## Figures and Tables

**Table 1:** Screened GSE datasets.

Tissue	Dataset	Platform	Normal	UC	Source
Mucosa	GSE47908	GPL570	15	20	https://www.ncbi.nlm.nih.gov/geo/query/acc.cgi?acc=GSE47908, accessed on 1 June 2021
Mucosa	GSE53306	GPL14951	12	16	https://www.ncbi.nlm.nih.gov/geo/query/acc.cgi?acc=GSE53306, accessed on 1 June 2021
Mucosa	GSE38713	GPL570	13	15	https://www.ncbi.nlm.nih.gov/geo/query/acc.cgi?acc=GSE38713, accessed on 6 June 2021

**Table 2:** KEGG pathway enrichment analysis of intersection genes and related DEGs.

ID	Term	Count	*p*-Value	Genes
hsa04060	Cytokine–cytokine receptor interaction	12	5.30 × 10^−6^	Interleukin 4 (IL4), C-C Motif Chemokine Ligand 24 (CCL24), Colony Stimulating Factor 3 Receptor (CSF3R), C-C Motif Chemokine Ligand 11 (CCL11), C-X-C Motif Chemokine Ligand 8 (CXCL8), C-C Motif Chemokine Ligand 20 (CCL20), Interleukin 1 Beta (IL1β), C-X-C Motif Chemokine Receptor 2 (CXCR2), C-X-C Motif Chemokine Ligand 1 (CXCL1), Colony Stimulating Factor 2 Receptor Beta Common Subunit (CSF2RB), C-X-C Motif Chemokine Ligand 2 (CXCL2), Interleukin 2 (IL2)
hsa05321	Inflammatory bowel disease (IBD)	7	1.60 × 10^−5^	IL4, Signal Transducer and Activator of Transcription 1 (STAT1), IL1β, Signal Transducer and Activator of Transcription 3 (STAT3), Nucleotide-binding Oligomerization Domain Containing 2 (NOD2), Forkhead Box P3 (FOXP3), IL2
hsa04621	NOD-like receptor signaling pathway	6	1.10 × 10^−4^	CXCL8, Caspase 5 (CASP5), IL1β, CXCL1, NOD2, CXCL2
hsa04062	Chemokine signaling pathway	9	1.70 × 10^−4^	CCL24, CCL11, CXCL8, STAT1, CCL20, STAT3, CXCR2, CXCL1, CXCL2
hsa04610	Complement and coagulation cascades	6	2.90 × 10^−4^	Serpin Family A Member 1 (SERPINA1), Plasminogen Activator, Urokinase (PLAU), Plasminogen Activator, Urokinase Receptor (PLAUR), Complement Component 4 Binding Protein Alpha (C4BPA), Complement Component 4 Binding Protein Beta (C4BPB), (Complement Factor B) CFB

**Table 3:** The ten most negatively correlated small molecule compounds screened by the CMap database.

RANK	CMap Name	Score	*n*	Enrichment	Percent Non-Null	Description
1	vorinostat	−1	12	0.304	0	HDAC antagonist
2	roxarson	−0.967	4	0.284	0	Animal growth promotant
3	wortmanni	−0.94	18	−0.459	55	PI3K inhibitor
4	sirolimus	−0.91	44	−0.175	20	Specific mTOR inhibitor
5	LY-294002	−0.903	61	−0.437	52	Synthetic broad-spectrum PI3K inhibitors
6	trichostatin A	−0.865	182	0.311	1	HDAC inhibitor
7	dequalinium chloride	−0.862	4	−0.328	25	Anti-microbial antiseptic agent
8	cinoxaci	−0.834	4	−0.434	50	Synthetic antibacterial agent
9	aminohippuric acid	−0.82	4	0.28	25	Diagnostic agent
10	amiodarone	−0.819	5	0.352	0	Antiarrhythmic medication

## Data Availability

The datasets generated during and/or analysed during the current study are available from the corresponding authors on reasonable request.

## Abbreviations

BP: Biological Process; C4BPA: Complement Component 4 Binding Protein Alpha; C4BPB: Complement Component 4 Binding Protein Beta; CASP5: Caspase 5; CFB: Complement Factor B; CXCL1: C-X-C Motif Chemokine Ligand 1 (also known as GRO Alpha); CXCL2: C-X-C Motif Chemokine Ligand 2; CXCL8: C-X-C Motif Chemokine Ligand 8 (also known as Interleukin 8); DEGs: Differentially Expressed Genes; FOXP3: Forkhead Box P3; GEO: Gene Expression Omnibus; GS: Gene Significance; GSEA: Gene Set Enrichment Analysis; IBD: Inflammatory Bowel Disease; IL1B: Interleukin 1 Beta; IL2: Interleukin 2; IL4: Interleukin 4; MAPK: Mitogen-Activated Protein Kinase; MM: Module Membership; NES: Normalized Enrichment Score; NF-κB: Nuclear Factor κB; NK: Natural Killer; NLR: NOD-like Receptor; NOD2: Nucleotide-binding Oligomerization Domain Containing 2; PPI: Protein–Protein Interaction; PLAU: Plasminogen Activator, Urokinase; PLAUR: Plasminogen Activator, Urokinase Receptor; SERPINA1: Serpin Family A Member 1; STAT1: Signal Transducer and Activator of Transcription 1; STAT3: Signal Transducer and Activator of Transcription 3Tfh: T Follicular Helper Cell; TNF-α: Tumor Necrosis Factor-α; UC: Ulcerative Colitis; WGCNA: Weighted Gene Co-Expression Network Analysis
